# Low birth weight and adverse pregnancy outcomes among women living with HIV and HIV-uninfected in Rwanda

**DOI:** 10.1371/journal.pone.0329312

**Published:** 2025-08-11

**Authors:** Natalia Zotova, Athanase Munyaneza, Gad Murenzi, Gallican Kubwimana, Adebola Adedimeji, Kathryn Anastos, Marcel Yotebieng, on behalf of the CA-IeDEA consortium

**Affiliations:** 1 Department of Medicine, Division of General Internal Medicine, Albert Einstein College of Medicine, Bronx, New York, United States of America; 2 Research for Development (RD Rwanda), Kigali, Rwanda; 3 Rwanda Military Hospital, Kigali, Rwanda; 4 Department of Social Sciences and Health Policy, Wake Forest University School of Medicine, Winston-Salem, North Carolina, United States of America; University of Zimbabwe Faculty of Medicine: University of Zimbabwe College of Health Sciences, ZIMBABWE

## Abstract

**Introduction:**

In utero exposure to HIV and/or antiretroviral therapy (ART) has been shown to be associated with stillbirth, preterm births, and low birth weight (LBW), but data from low-resource, high- HIV-burden settings remain limited. This study describes adverse pregnancy outcomes among Rwandan women living with HIV (WLWH) and HIV-uninfected women and examines associations between HIV, ART timing, and LBW.

**Methods:**

This retrospective cohort study used antenatal care, delivery, and PMTCT registry data from the Central Africa International Epidemiology Databases to Evaluate AIDS (CA-IeDEA). Women with documented HIV status and recorded birth weights were included. Adverse outcomes were defined as LBW (<2,500 g), stillbirth, and preterm birth (<37 weeks gestation). Logistic regression was used to assess associations between maternal HIV status, ART timing, and LBW, adjusting for relevant covariates.

**Results and discussion:**

Among 10,191 women with known HIV status and babies’ birth weights, 12.7% (n = 1,293) were WLWH. There were 47 stillbirths (0.5%) and 70 preterm births (0.7%). Among 10,037 term births, 366 (3.6%) were LBW. WLWH had significantly higher rates of stillbirth (0.6% vs. 0.4%, p < 0.05) and LBW (6.5% vs. 2.9%, p < 0.001) compared to HIV-uninfected women; preterm birth rates did not differ significantly. The adjusted odds of LBW among WLWH were 1.61 (95% CI: 1.08, 2.39), controlling for marital status, primigravidae, and maternal weight at admission. Among WLWH (n = 1,274), ART initiation prior to pregnancy was associated with 50% lower odds of LBW after adjusting for age and WHO stage.

**Conclusions:**

Even among relatively healthy uncomplicated pregnancies in low-risk delivery settings and universal ART, WLWH experienced significantly higher rates of stillbirth and LBW. Among WLWH, initiation of ART prior to current pregnancy had a protective effect against LBW. This underscores the importance of early HIV diagnosis and initiation of ART.

## Introduction

Following the 2010 World Health Organization (WHO) guidelines [1], some countries, for pragmatic reasons, adopted the Option B – triple antiretroviral therapy (ART) for all pregnant women living with HIV (WLWH) for the prevention of mother-to-child transmission (PMTCT) of HIV starting as soon as possible during pregnancy and continuing through the end of all breastfeeding. In its 2013 updated guidelines the WHO recommended that for PMTCT, lifelong ART be expanded to all pregnant and breastfeeding WLWH regardless of CD4 count (Option B+) [2]. By 2015, 91% of the 1.1 million women globally receiving ART to prevent mother-to-child transmission were on lifelong therapy [3]. The increased availability of ART for pregnant women has dramatically reduced mother-to-infant transmission of HIV [4]. One consequence of this success in PMTCT is that millions of HIV-uninfected infants are exposed in utero—and through breastfeeding up to 2 years of age—to both HIV and multiple antiretroviral drugs (ARVs), with limited data on their long- term safety [5].

Studies conducted before the Option B/B + era showed that children who are HIV-exposed and uninfected (CHEU) have a higher risk of morbidity and mortality compared with children born to HIV-negative mothers, [6–12]. Advanced maternal HIV disease during pregnancy has been linked to elevated morbidity and mortality in CHEU [13,14], particularly due to increased risks of preterm birth (PTB) risks and associated LBW. Higher concentrations of HIV in the placenta during fetal development have been inversely correlated with birth weight [15].

By reducing morbidity in mothers with HIV [16–18], universal ART under Option B/B + has the potential to indirectly reduce morbidity and mortality risks among CHEU. However, in utero exposure to ART may also have adverse long-term health effects. Such exposure increases the risk of prematurity and LBW [19–24], both of which are major contributors to neonatal death [25,26]. Moreover, LBW infants face a heightened risk of neurodevelopmental impairment, including cerebral palsy [27,28], impaired lung function and respiratory morbidity [29], and an increased likelihood of adult-onset diseases such as type II diabetes mellitus, hypertension, and cardiovascular disease [30]. A meta-analysis of 43 studies from 21 countries estimated the odds ratio of LBW associated with maternal HIV infection as 1.7 (95% CI: 1.6, 1.8) [31], a figure that has remained largely unchanged since 1989. However, most studies included in this analysis were conducted before the widespread adoption of universal ART for pregnant women.

Given the near-universal ART coverage among pregnant WLWH and the growing number of CHEU worldwide [32], updated data on adverse pregnancy outcomes – including LBW – are needed to inform policy and interventions. For example, the PROMISE trial, conducted in seven countries across East and Southern Africa and India, compared three ART regimens used by WLWH during pregnancy. The study found that tenofovir disoproxil fumarate (TDF)-based regimens were associated with higher risks of adverse pregnancy outcomes and LBW compared to zidovudine (ZDV) regimens [33]. The widespread adoption of TDF-based regimens in Central Africa highlights the need to closely monitor their impact on CHEU.

Any health problems associated with HIV or ART exposure among uninfected children have significant public health implications. Yet, evidence on the association between in utero HIV and/or ART exposure in routine care and LBW in low-income, high HIV-burden settings remains limited. This study addresses this gap by using retrospectively collected data to describe stillbirths, preterm births, and LBW among both WLWH and HIV-uninfected women in Rwanda – and to assess the association of HIV and/or ART exposure with outcomes among singleton live births.

## Materials and methods

### Study design and setting

This retrospective cohort study used data collected in Rwanda within the Central Africa International Epidemiology Databases to Evaluate AIDS (https://ca-iedea.org/). CA-IeDEA is part of an international https://www.iedea.org/ established in 2005 by the U.S. National Institute of Allergy and Infectious Disease (NIAID) to address high priority HIV and AIDS research questions. CA-IeDEA currently includes 22 sites in Burundi, Cameroon, the Democratic Republic of Congo (DRC), the Republic of Congo, and Rwanda. Rwanda, a country in Central Africa with a population of more than 13 million [34] people, has been implementing Option B+ since 2012 proving lifelong ART for pregnant and breastfeeding WLWH. Rwanda has one of the most successful ART programs in the world, with high rates of HIV diagnosis and ART coverage, along with high rates of retention and viral suppression [35]. In 2019, 97% of pregnant WLWH in Rwanda received ART for PMTCT [36]. Universal ART availability for pregnant WLWH in Rwanda has contributed to a significant reduction of mother-to-child HIV transmission to less than 2% since implementation of Option B+ [36], resulting in a growing number of CHEU [37,38].

### Data collection and population

From February 1, 2018 to September 30, 2021, trained research nurses visited each of the ten health facilities participating in CA-IeDEA with antenatal care, delivery and PMTCT services. With the help of the facility, they accessed antenatal care (ANC), delivery and PMTCT registries and extracted routinely collected data from those registries. Study data were then entered to and managed using REDCap electronic data capture tools [39] hosted at The Ohio State University. Data were accessed for research purposes on October 30, 2021. During data collection, AM and GM had access to information that could identify individual participants. The dataset was stripped of identifiers prior to the analysis.

Data from antenatal care and delivery registries were manually extracted and linked across the two registries for all women who received care in the facility starting November 2010, when Rwanda implemented Option B. In addition to ANC and delivery registries, HIV and PMTCT data were obtained from mother-infant pair registries in PMTCT services. In Rwanda’s health care settings, women having potentially complicated or high-risk pregnancies are mostly transferred to district hospitals for ANC services and childbirth. Our sample thus included data from ANC, delivery, and PMTCT registries on generally healthier uncomplicated pregnancies. Data were obtained retrospectively from all CA-IeDEA affiliated health facilities; a consent waiver was obtained for this secondary analysis of existing data. The study was approved by the Rwanda National Ethics Committee and the Ohio State University Institutional Review Board.

Eligible participants were women with a documented HIV test result in ANC registries and birth weight in delivery registries. Doctors or registered nurses used scales in maternity wards to measure birth weight and recorded it along with gestational age in delivery registries. Adverse pregnancy outcomes assessed also included stillbirth (a baby who dies after 28 weeks of pregnancy, but before or during birth), and preterm birth (gestational age < 37 weeks).

### Outcomes of interest and variable definitions

The primary outcome of interest was low birth weight (<2,500 grams). Other outcomes assessed were stillbirth and preterm birth. The main exposures were mother’s HIV status and timing of ART initiation, for WLWH only categorized as “during current pregnancy” vs. “prior to current pregnancy”. Independent variables considered included women’s age (categorized into three groups: ≤ 24, 25–34, and ≥35 years), marital status (married/cohabiting vs. single/divorced/separated/widowed), mother’s weight at the time of delivery (categorized into three groups <60, 60–64, and ≥65 kg), and primigravidae status (no vs. yes). All infants born to a WLWH were considered to have been exposed in-utero to HIV and/or ART, mostly TDF + Lamivudine (3TC) + Neviparine (NVP) or TDF + 3TC + Efivarenz (EFV) regimens. For WLWH, the World Health Organization (WHO) stage of HIV infection (1–4) was also considered as covariate.

### Statistical analyses

Primary and secondary outcomes of interest and sociodemographic and clinical factors included in the models were summarized using proportions, means or median as appropriate. A Chi-square test was used instead to compared the proportion of adverse outcomes across exposure categories. Logistic regression models were used to estimate the odd ratios (ORs) and 95% confidence intervals (95%CI) assessing the strength of the association between LBW and mother’s HIV status or timing of ART. The frequency of stillbirths and preterm did not allow for multivariate analyses. Analyses were stratified by preterm birth only and term plus preterm (whole sample) given the known association of preterm birth and ART [24]. Variates that were found to be associated with LBW at p < 0.2 in bivariable models were included in the multivariable models. All statistical analyses were conducted using Stata Version 16.0. [40].

## Results

**Fig 1:**
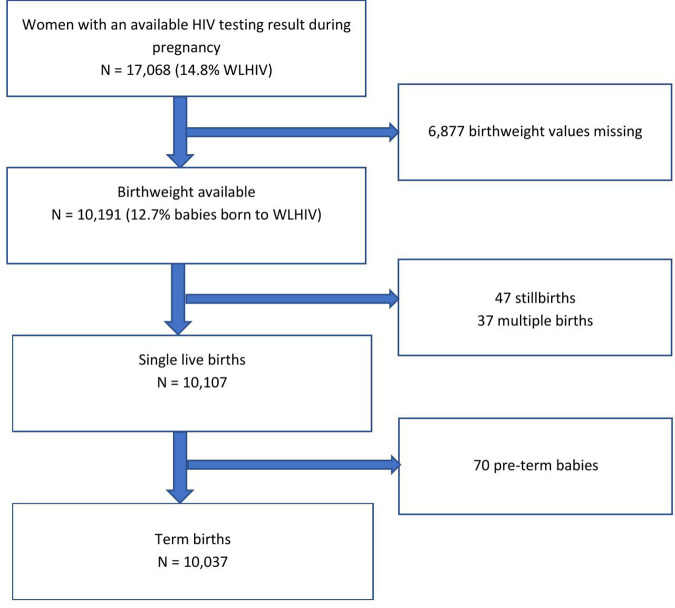
Inclusion flowchart.

A total of 17,068 women had an available HIV testing result during pregnancy (Fig 1). Of these, 10,191 women had a known HIV status and documented birth weight for their babies. Among these 10,191 women, 12.7% (n = 1,293) were WLWH. One-third of women (n = 2,530) were aged 24 or less (Table 1). The majority of women (86.3%, n = 8,790) were married or cohabiting and had been pregnant before (non-primigravida) (74.2%, n = 7,558). At the time of delivery, about one-third of women (30.9%, n = 3,155) weighed 60 kilograms or less, 24.5% (n = 2,496) weighed 60–64 kg, and 35.2% (n = 3,583) weighed 65 and more kilograms. Virtually all WLWH (n = 1,106) were using ART. Of WLWH with a documented date of the ART start (n = 813), more than half started the medication prior to current pregnancy (57.9%, n = 476). Most WLHW (56.9%, n = 736) had WHO HIV stage 1 and 31.8% (n = 411) had a missing WHO stage value (Table 1).

**Table 1:** Socio-demographic and clinical characteristics of 10,191 women, who had HIV test results and babies’ birth weight available.

	WLWH (n = 1,293, %)	Women without HIV (n = 8,898)	Total (n = 10,191)
HIV status			
Negative	0	8,898	8,898 (87.3)
Positive	1,293	0	1,293 (12.7)
Missing	0	0	0 (0)
Age			
<=24	126 (9.7)	2,404 (27)	2,530 (24.8)
24–34	371 (28.7)	3,720 (41.8)	4,091 (40.1)
35+	159 (12.3)	1,030 (11.6)	1,189 (11.7)
Missing	637 (49.3)	1,744 (19.6)	2,381 (23.4)
Marital status			
Single	178 (13.8)	853 (9.5)	1,031 (10.1)
Married/cohabiting	1,071 (82.8)	7,719 (86.8)	8,790 (86.3)
Missing	44 (3.4)	326 (3.7)	370 (3.6)
Weight at admission, kg			
<60	284 (22)	2,871 (32.3)	3,155 (30.9)
60–64	174 (13.5)	2,322 (26.1)	2,496 (24.5)
65+	281 (21.7)	3,303 (37.1)	3,583 (35.2)
Missing	554 (42.8)	403 (4.5)	957 (9.4)
Primigravida			
No	1,102 (85.2)	6,456 (72.5)	7,558 (74.1)
Yes	190 (14.7)	2,437 (27.4)	2,627 (25.8)
Missing	1 (0.1)	5 (0.1)	6 (0.1)
ART used during pregnancy			
No	70 (5.4)	NA	NA
Yes	1,123 (86.9)	NA	NA
Missing	100 (7.7)	NA	NA
ART started		NA	NA
During current pregnancy	346 (26.8)	NA	NA
Prior to current pregnancy	476 (36.8)	NA	NA
Missing	471 (36.4)	NA	NA
WHO HIV stage			
1	736 (56.9)	NA	NA
2	93 (7.2)	NA	NA
3	47 (3.6)	NA	NA
4	6 (0.5)	NA	NA
Missing	411 (31.8)	NA	NA
*Pregnancy outcomes*			
Stillbirth			
No	601 (46.5)	7,048 (79.2)	7,649 (75.1)
Yes	8 (0.6)*	39 (0.4)	47 (0.5)
Missing	684 (52.9)	1,811 (20.4)	2,495 (24.4)
Low birth weight (<2500 gram), all single live births			
No	1,194 (93.3)	8,547 (96.8)	9,741 (96.4)
Yes	86 (6.7)***	280 (3.2)	366 (3.6)
Missing	0 (0)	0 (0)	0 (0)
Preterm births (<37 weeks)			
No	725 (56.6)	8,251 (93.5)	8,976 (88.8)
Yes	6 (0.5)	64 (0.7)	70 (0.7)
Missing	549 (42.9)	512 (5.8)	1,061 (10.5)
Low birth weight (<2500 gram), single live births, term babies			
No	1,191 (93.5)	8,507 (97.1)	9,698 (96.6)
Yes	803 (6.5)***	256 (2.9)	339 (3.4)
Missing	0 (0)	0 (0)	0 (0)

* Chi-square test of differences between adverse pregnancy outcomes among WLWH and women without HIV; significance levels: + p < 0.1; * p < 0.05; ** p < 0.01; *** p < 0.001

### Pregnancy outcomes

Of 10,191 pregnancies, 47 (0.5%) were stillbirths, 37 (0.3%) – multiple births, and 10,107 (99.2%)- single live births, respectively (Fig 1). Of 10,107 single live births, 10,037 (99.3%) resulted in term and 70 (0.7%) – in pre-term births. Among 10,037 term singletons, 339 (3.4%) weighed less than 2,500 g. WLWH had a significantly higher proportion of LBW babies (6.5% vs 2.9%, p < 0.001) and stillbirth (0.6% vs. 0.4%, p = 0.02), but preterm births did not differ significantly between WLWH and women without HIV (0.7% vs 0.5%, p = 0.9).

### Effect of mother’s HIV status on LBW

In bivariate analyses of single term births (n = 10,037), WLWH had significantly higher odds of having low weight babies compared to HIV-negative women (OR 2.32; 95% CI 1.79, 2.99; p < 0.001) (Table 2). Among potential confounders considered, primigravidae status was also associated with higher odds of LBW (OR 1.79; 95% CI 1.43, 2.23; p < 0.001). Odd ratios of LBW were significantly lower among married/cohabiting women (OR 0.68; 95% CI 0.5, 0.93; p < 0.05) compared to single women. Higher body weight at admission to maternity clinics was associated with significantly lower odds of having low weight babies (60–64 kg vs. 60 or less (OR 0.48; 95% CI 0.35, 0.66; p < 0.001) and 65 + kg vs. 60 or less (OR 0.36; 95% CI 0.27, 0.49; p < 0.001).

**Table 2:** Bivariate and multivariable associations between women’s HIV status, socio-demographic and clinical characteristics, and low birth weight.

	Single live births, term babies (n = 10,037)			All single live births (n = 10,107)		
	N*	OR (95% CI)	aOR (95% CI)	N	OR (95% CI)	aOR (95% CI)
HIV status						
Negative	8,763			8,827		
Positive	1,274	2.32 (1.79, 2.99)***	1.61 (1.08, 2.39)*	1,280	2.2 (1.71, 2.82)***	1.62 (1.11, 2.36)**
Age						
<=24	2,492			2,514		
25–34	4,032	0.81 (0.61, 1.09)		4,063	0.79 (0.6,1.04)^+^	
35+	1,159	0.9 (0.6, 1.35)		1,168	0.85 (0.57,1.25)	
Marital status						
Single	1,013			1,018		
Married/cohabiting	8,661	0.68 (0.5, 0.93)*	0.74 (0.52, 1.05)	8,724	0.71 (0.52,0.96)*	0.78 (0.56,1.09)
Weight at admission, kg						
<60	3,098			3,127		
60–64	2,456	0.48 (0.35, 0.66)***	0.46 (0.33, 0.63)***	2,476	0.51 (0.38, 0.68)***	0.49 (0.37, 0.67)***
65+	3,533	0.36 (0.27, 0.49)***	0.4 (0.29, 0.53)***	3,553	0.36 (0.27, 0.48)***	0.39 (0.29, 0.53)***
Primigravida						
No	7,445			7,492		
Yes	2,586	1.79 (1.43, 2.23)***	1.89 (1.46, 2.45)***	2,609	1.8 (1.45,2.24)***	1.88 (1.47, 2.41)***

* Frequencies might not add up to the total for the category because of missing data;

** Significance levels: + p < 0.1; * p < 0.05; ** p < 0.01; *** p < 0.001

In a multivariable model including marital status, mother’s weight at delivery, and primigravidae status, positive HIV status remained significantly associated with LBW (aOR 1.61; 95% CI 1.08, 2.39; p < 0.05).

When the analytical sample was expanded to all single term and preterm births (n = 10,107), the associations between mother’s HIV status and LBW remained statistically significant in bivariate (OR 2.2; 95% CI 1.71, 2.82; p < 0.001) and multivariable analyses (aOR 1.62; 95% CI 1.11, 2.36; p < 0.01) (Table 2).

### Effect of timing of ART on LBW

Among WLWH (n = 1,274), in bivariate analyses of single term births, the ART start prior to current pregnancy, though not statistically significant, was associated with a lower odds of LBW babies compared to women who started ART during current pregnancy (OR 0.9; 95%CI 0.52, 1.56). Compared to WHO stage 1, mothers in WHO stage 2 had higher odds of LBW (OR 1.7; 95% CI 0.83, 3.48; p = 0.15). Of other potential confounders, mother’s higher body weight at was associated with significantly lower odds of having low weight babies (60–64 kg vs. 60 or less (OR 0.32; 95% CI 0.11, 0.95; p < 0.05) and 65 + kg vs. 60 or less (OR 0.4; 95% CI 0.17, 0.94; p < 0.05) (Table 3).

**Table 3:** Bivariate and multivariable associations between socio-demographic and clinical characteristics of WLWH, and LBW.

	Single live births, term babies (n = 1,274)			All single live births (n = 1,280)		
	N*	OR (95% CI)	aOR (95% CI)	N	OR (95% CI)	aOR (95% CI)
Age						
<=24	125			125		
25–34	362	0.81 (0.35, 1.9)		366	0.85 (0.36, 1.97)	
35+	153	0.6 (0.2, 1.77)		154	0.7 (0.25, 1.98)	
Marital status						
Single	175			175		
Married/cohabiting	1,057	1.37 (0.67, 2.79)		1,063	1.42 (0.7, 2.89)	
Weight at admission, kg						
<60	275			279		
60–64	170	0.32 (0.11, 0.97)*	0.68 (0.2, 2.34)	172	0.37 (0.14, 0.99)*	0.61 (0.18, 2.05)
65+	275	0.4 (0.17, 0.94)*	0.58 (0.18, 1.86)	275	0.37 (0.16, 0.85)*	0.53 (0.17, 1.63)
Primigravida						
No	1,088			1,092		
Yes	185	1.44 (0.82, 2.55)		187	1.37 (0.78, 2.41)	
ART initiated						
During current pregnancy	342			356		
Prior to current pregnancy	476	0.9 (0.52, 1.56)	0.49 (0.18, 1.39)	602	0.89 (0.52, 1.52)	
WHO HIV stage						
1	724			727		
2	93	1.7 (0.83, 3.48)	3.26 (1.1, 9.58)*	93	1.67 (0.81, 3.42)	3.79 (1.06, 13.47)*
3	45	1.76 (0.66, 4.67)	2.28 (0.47, 11)	46	2.08 (0.84, 5.13)	3.31 (0.63, 17.04)
4	6	empty	empty	6	empty	empty

* Frequencies might not add up to the total for the category because of missing data;

** Significance levels: + p < 0.1; * p < 0.05; ** p < 0.01; *** p < 0.001

In the multivariable analysis, adjusting for mother’s weight, WHO stage, and mothers WHO stage, ART initiation prior to current pregnancy tended towards lower odds of LBW (aOR 0,49; 95% CI 0.18, 1.39; p = 0.18) but was not statitically significant. Expansion of the analytical sample to all term and preterm births showed similar results.

## Discussion

In this study, we used data from a large sample of Rwandan women to describe pregnancy outcomes and to examine factors associated with LBW including mother’s HIV status and/or ART use. Globally, the prevalence of preterm birth varies by country and it is estimated to be about 12% in sub-Saharan Africa [41]. Overall, with <1% of preterm birth and stillbirth, the prevalence of poor pregnancy outcomes was relatively low in our sample irrespective of HIV status. Moreover, about 14% of livebirths in SSA are estimated to be LBW [42]. The 3.5% prevalence of LBW in our sample is also lower than the 6.9% reported in the Rwanda recent national demographic health survey [43]. This may be a result of the fact that all 10 participating HIV clinics were in health centers. In the Rwandan pyramidal health system, only uncomplicated pregnancies and deliveries are handled at the level of the health centers. Women with preterm labor, who are more likely to deliver preterm and LBW are referred to the district hospitals.

Consistent with a recent systematic review [44], WLWH in our sample have higher prevalence of stillbirths and LBW infants. In a meta-analysis published before May 2015, Xia et al. [31] found that maternal HIV infection was significantly associated with both LBW and preterm delivery. However, they also found that ART did not significantly change the associations of maternal HIV exposure with LBW and preterm delivery. Although our odds ratio of 1.61 measuring the size of the association of HIV/ART with LBW is slightly lower than the 1.73, reported by Xia et al, its 95% CI overlaps their pooled estimate. Our findings are consistent with a recent registry study from Malawi. Chamanga et al. [45] compared adverse birth outcomes among WLWH and HIV-negative women delivering in high (a referral hospital) and low risk (primary healthcare facilities) settings. They showed that rates of LBW and preterm births are significantly higher among WLWH compared to HIV-negative women and those differences are more pronounced in high-risk settings than in low-risk PHC facilities. This aligns with this study’s findings that showed that even in a sample of relatively healthy uncomplicated pregnancies delivered in low-risk settings, WLWH still had significantly higher rates of stillbirth and low weight babies compared to women without HIV.

Among WLWH, initiation of ART prior to pregnancy tended to be protective of LBW, but was not statistically significant. A recent systematic review has pointed out on the protective role of ART on adverse pregnancy outcomes including LBW. The review found that WLWH receiving ART had a significantly decreased risk of preterm birth and low weight babies compared to ART-naïve WLWH [46]. Our study findings align with this global evidence. Without protection, offered by ART, HIV-related inflammation affects fetal development to a greater extent and may lead to LBW and other adverse pregnancy outcomes [19,46].

Our study has some limitations. As discussed above, our sample may be biased towards healthier pregnancies and thus better pregnancy outcomes due to the systematic referral of any potentially complicated pregnancy/delivery to district hospitals. These complicated pregnancies may be independently associated with poor outcomes. This in turn may explain the relatively low prevalence of poor pregnancy outcomes in our sample despite the large number of women and births. Retrospectively linking datasets from ANC and delivery registries allowed for rich data from a large cohort of women. However, data missingness was high for some key variables and guided our decision to not include key important variables like women’s education, occupation and income. These factors could have affected the socioeconomic and nutritional statuses of women during pregnancy.

Despite these limitations, this study has several strengths. It uses data from a large cohort of women in Rwanda, a country that has implemented Option B+ providing lifelong ART to all pregnant WLWH since 2012 and thus allowing us to revisit the association between exposure to HIV and ART during pregnancy and pregnancy outcomes in this era of universal ART. To our knowledge, there have been no studies assessing the association between *in utero* HIV and/or ART exposure and LBW in Rwanda and only a few similar studies in other sub-Saharan countries [33,47,48]. This study contributes to the literature and advances in understanding of the effects of HIV and/or ART exposure during pregnancy on birth outcomes, and birth weight, which have important implications for infants’ growth, development, morbidity and mortality in the long-term perspective.

## Conclusions

Even in the era of universal ART, Rwandan WLWH have higher risks of having low weight infants compared with HIV-uninfected women. Initiation of ART prior to current pregnancy was shown to have a non-statistically protective effect against LBW. This underscores the importance of targeted prenatal care interventions that address maternal health including early HIV diagnosis and initiation of ART to reduce the risk of low birth weight.
